# Journal of Experimental & Clinical Cancer Research reviewer acknowledgement 2012

**DOI:** 10.1186/1756-9966-32-19

**Published:** 2013-04-22

**Authors:** Mauro Castelli

**Affiliations:** 1 "Istituto Regina Elena" National Cancer Institute, Rome, Italy

## Abstract

**Contributing reviewers:**

The editor of *Journal of Experimental & Clinical Cancer Research* would like to thank all our reviewers who have contributed to the journal in Volume 31 (2012).

## 

Michael Abend

Germany

Byeong-Cheol Ahn

Korea South

Sheikh Mohammad Fazle Akbar

Japan

Eiman Aleem

Sweden

Paolo Amerio

Italy

Jie An

United States of America

Vibeke Andersen

United Kingdom

Hiroyoshi Ariga

Japan

V Satya Suresh Attili

India

Georges Baffet

France

Enzo Berardesca

Italy

Lothar Bergmann

Germany

Vikas Bhardwaj

United States of America

Guido Bocci

Italy

Laura Bonanno

Italy

Gianluca Bossi

Italy

Georg Breier

Germany

Emilio Bria

Italy

Jan Bussink

Netherlands

Valentina Caracciolo

United States of America

Jason Carroll

United Kingdom

Pradeep Chaluvally Raghavan

United States of America

Yoon Soo Chang

Korea South

Zhe Chang

United States of America

Shuzhen Chen

United States of America

Xian-Ming Chen

United States of America

Zihua Chen

China

Wen-Fang Cheng

Taiwan

Vilai Chentanez

Thailand

Chi Hin Cho

China

William CS Cho

Hong Kong

Yen-Hung Chow

Taiwan

Mara Cirone

Italy

PierFranco Conte

Italy

Alfredo Conti

Italy

Pierre Cordelier

France

Giovanni Corso

Italy

Paul Cottu

France

Bingbing Dai

United States of America

Enrique de Alava

Spain

Juan Carlos de Vicente

Spain

Hongxin Deng

China

Jing-yu Deng

China

Marc Denis

France

Danilo Di Bona

Italy

Cherubino Di Lorenzo

Italy

Song-Ze Ding

China

Thomas Dittmar

Germany

Jessica Donington

United States of America

Gabriella D'Orazi

Italy

Wenrui Duan

United States of America

Dan Duda

United States of America

Mohan Edirisinghe

United Kingdom

Karin Ekstrom Smedby

Sweden

Francesco Facciolo

Italy

Rita Falcioni

Italy

Yue-Zu Fan

China

Dianchun Fang

China

Weiyi Fang

China

Ambrogio Fassina

Italy

Fei Fei

United States of America

Falko Fend

Germany

Glenn Flux

United Kingdom

Marco Folini

Italy

Jing Fu

United States of America

Jianmin Fu

China

Toshiyoshi Fujiwara

Japan

Amy Fulton

United States of America

Mayuko Furuta

Japan

Rossella Galati

Italy

Apar Kishor Ganti

United States of America

Lina Gao

United States of America

Qinglei Gao

China

Isabella Gashaw

Germany

Margaret Gates

United States of America

Paramita Ghosh

United States of America

Patrizio Giacomini

Italy

Constantinos Giaginis

Greece

Elena Giglia

Italy

Anna Gillio-Tos

Italy

Antonio Giordano

Italy

Glenda Gobe

Australia

Zihua Gong

United States of America

Laura Gramantieri

Italy

Aleksandar Grgic

Germany

Yan Gu

China

Antonino Guerrisi

Italy

Ian Guest

United States of America

Junming Guo

China

Cheng Guo

China

Joerg Haier

Germany

Subrata Haldar

United States of America

Inn-Oc Han

Korea South

Jitti Hanprasertpong

Thailand

Lorna Harries

United Kingdom

Takahiro Hasebe

Japan

M. Raschid Hoda

Germany

Ann Hopkins

Ireland

Yingchun Hou

China

Wenwei Hu

United States of America

Xiaotong Hu

China

Ai Hua

China

Bo Huang

China

Jianfei Huang

China

Yulun Huang

China

Mikito Inokuchi

Japan

Yasushi Ishida

Japan

Sumiya Ishigami

Japan

seiji isonishi

Kiribati

Jin Jen

United States of America

Hernandez Jesus M

Spain

Gautam Jha

United States of America

Sheng-Yu Jin

United States of America

Noor Kamil

Pakistan

Manabu Kawada

Japan

Nayoung Kim

Korea South

Maggie Kirkman

Australia

Jan Kitajewski

United States of America

Takako Kizaki

Japan

Barbara Klughammer

Switzerland

Katsumi Kobayashi

Japan

Takashi Kumagai

Japan

Sanath Kumar

United States of America

Gopal Kundu

India

Kaoru Kusama

Japan

Jane Lane

United Kingdom

Tanja Langsenlehner

Austria

Chung Lee

United States of America

Won-Ha Lee

Korea South

Jae Cheol Lee

Korea South

Kwang-Ho Lee

Korea South

Carlo Leonetti

Italy

Chiara Lestuzzi

Italy

Xin Li

China

Yan Li

China

Jing LI

United States of America

Xu Li

China

Fengzhi Li

United States of America

Jie Liang

United States of America

Weng Lihong

China

Jae-Young Lim

Korea South

Dechen Lin

United States of America

Chi-Iou Lin

United States of America

Qi Ling

China

Xudong Liu

China

Xi Liu

United States of America

Chien-Ying Liu

Taiwan

Yong-Yu Liu

United States of America

Zhi Liu

United States of America

Yuguang Liu

China

Jin-Hwang Liu

Taiwan

Jinsong Liu

United States of America

Caigang Liu

China

Patricia Lorenzo

United States of America

Yong Lu

United States of America

Shen Lu

China

Yuanzhi Lu

United States of America

Jun Lu

China

Bo Lu

United States of America

Hong Lok Lung

Hong Kong

Teng Ma

United States of America

Yanlei Ma

China

Steen Madsen

United States of America

Sarmila Majumder

United States of America

Shyamali Mandal

United States of America

Yoshimasa Maniwa

Japan

Bruno Märkl

Germany

Isao Matsui-Yuasa

Japan

Toshiharu Matsumoto

Japan

Koji Matsuo

United States of America

Annelies Mavinkurve

Netherlands

John McKolanis

United States of America

Yuping Mei

United States of America

Michele Milella

Italy

Anna Maria Mileo

Italy

Jiao Min

China

Mei Ming

United States of America

Koichi Miyake

Japan

Yasuo Miyoshi

Japan

Shusaku Mizukami

Japan

Elena Moiseeva

United Kingdom

Yuseok Moon

Korea South

Eiichi Morii

Japan

Laura Moro

Italy

Catherine Muller

France

Veronika Müller

Hungary

Chisato Nakada

Japan

Masataka Nakamura

Japan

Pediconi Natalia

Italy

Jens Neumann

Germany

Shozo Nishida

Japan

Ken Olaussen

France

Shinji Osada

Japan

Makoto Osanai

Japan

Jing Pan

China

Sofia Pavanello

Italy

Edward Peres

United States of America

Nicola Pimpinelli

Italy

Sharon Prince

South Africa

Alessandro Provenzani

Italy

Andika Putra

Japan

Li Qian

China

Ai-Rong Qian

China

Kumaraguruparan Ramasamy

United States of America

Daniel Ramos

United States of America

Claudia Rossig

Germany

Daniel Ruan

United States of America

Giuseppe Russo

United States of America

Mohit Sachdeva

United States of America

Kennichi Satoh

Japan

Joshua Schiffman

United States of America

Klaus Schmitz

Germany

Joachim Schneider

Germany

Kumar Selvarajoo

Japan

Prem Seth

United States of America

Xiaobin Shang

China

Jian-Yong Shao

China

Huiyong Shen

China

Jin-Yuan Shih

Taiwan

Takashi Shingu

United States of America

Bhuminder Singh

United States of America

Garima Singhal

United States of America

Ondrej Slaby

Czech Republic

Yong Song

China

Zhongchen Song

China

Jeff Spees

United States of America

Sonja E. Steigen

Norway

Ulrike Stein

Germany

Sabrina Strano

Italy

Hiroshi Suemizu

Japan

Takeshi Sugimoto

Japan

Haruhiko Sugimura

Japan

Zhaoyu Sun

United States of America

LuZhe Sun

United States of America

Yanan Sun

China

Marek Svoboda

Czech Republic

Masayuki Takeda

Japan

Seiichi Takenoshita

Japan

Hiroya Takeuchi

Japan

Benjamin Tan

United States of America

Toshiyuki Tanaka

Japan

Woan-Yuh Tarn

Taiwan

Tommaso Tartaglione

Italy

Shenghuo Tian

United States of America

Patrizia Tosi

Italy

Antonella Tosti

United States of America

Kazunori Tsukuda

Japan

Shuiping Tu

China

Daisuke Uchida

Japan

Kedar Vaidya

United States of America

Shaogui Wan

United States of America

Jiansheng Wang

China

Shulin Wang

China

Yan Wang

United States of America

Zefeng Wang

United States of America

Jun Wang

China

Shui Wang

China

Yucai Wang

United States of America

Lei Wang

United States of America

Ke Wang

China

yingqun wang

United States of America

Anxun Wang

China

Michael Weller

Germany

Vicki Whitehall

Australia

Marcus Wiedmann

Germany

Yantao Wu

China

Xin Wu

United States of America

Yi-Long Wu

China

Xiaosheng WU

United States of America

Ming-Heng Wu

Taiwan

Qiang Xiao

China

Chengzhi Xie

United States of America

Zhaoquan Xing

China

Qiwu Xu

American Samoa

Li Xu

United States of America

Jia Xu

United States of America

Ningzhi Xu

China

Chuan-shan Xu

China

Wei Huan Yan

China

Libo Yan

United States of America

Chia-Ron Yang

Taiwan

Hanshuo Yang

China

Ya-Chien Yang

Taiwan

Liangqing Yao

China

Dengfu Yao

China

Jun Yao

China

Thomas Yau

Hong Kong

Zhong Ye

United States of America

Lu Yicheng

China

Huquan Yin

United States of America

Zhirong Yin

United States of America

Qi Yin

United States of America

Yongping You

China

Ke Zen

China

Yushan Zhang

United States of America

Hong Zhang

United States of America

Shiwu Zhang

China

Zhenwei Zhang

United States of America

Xuemei Zhang

United States of America

Yong Zhang

United States of America

Kejun Zhang

China

Tao Zhang

United States of America

Xuejun Zhao

United States of America

Po Zhao

China

Liduan Zheng

China

Limin Zheng

China

Tianhua Zhou

China

Genyin Zhou

China

Li Zhou

China

Bing Zhou

United States of America

Lingling Zhu

China

Yang Zong

United States of America

